# Prevention and Management of Carboplatin Nephrotoxicity

**DOI:** 10.3390/cancers18172801

**Published:** 2026-08-28

**Authors:** Renato A. Caires, Elerson C. Costalonga, Verônica Torres Costa e Silva

**Affiliations:** 1 Sao Paulo State Cancer Institute, Division of Nephrology, University of Sao Paulo School of Medicine, Sao Paulo 01246-000, Brazil; r.caires@hc.fm.usp.br; 2 Division of Nephrology, Renal Vida Association, Blumenau 89010-500, Brazil; elerson.costalonga@renalvida.org.br; 3 Laboratório de Investigação Médica (LIM-16), Division of Nephrology, University of Sao Paulo School of Medicine, Sao Paulo 01246-903, Brazil

**Keywords:** carboplatin, nephrotoxicity, glomerular filtration rate, acute kidney injury, chronic kidney disease, Calvert formula, dose adjustment

## Abstract

Carboplatin, a second-generation platinum-based therapy, is widely indicated for first-line treatment of selected solid tumors. Furthermore, it represents a well-established alternative to cisplatin in populations characterized by chronic kidney disease (CKD) or clinical frailty. The renal profile of carboplatin toxicity is most commonly linked to hypomagnesemia, and less frequently to acute kidney injury (AKI) or a persistent decline in GFR. While hypomagnesemia is the most frequent renal adverse effect, severe episodes that impact anticancer treatment are uncommon. Reductions in GFR carry significant clinical implications because they directly affect carboplatin dosing and drive cumulative hematologic toxicity that leads to adverse clinical events, which may be more relevant in older and sarcopenic patients. Carboplatin toxicity is highly dependent on systemic exposure and is quantified by the area under the concentration–time curve (AUC). Inaccurate estimation of GFR, particularly when using the Cockcroft–Gault (CG) equation, frequently leads to GFR overestimation and carboplatin overdose. Thus, precise dose adjustment according to GFR is of the utmost importance. In this review, we examine the renal complications associated with carboplatin, including the reported incidence and clinical patterns of kidney injury, with particular emphasis on dose-adjustment strategies based on GFR, encompassing patients with CKD and those receiving dialysis, as well as practical considerations for the prevention and management of carboplatin nephrotoxicity.

## Simple Summary

Carboplatin is widely used as a first-line therapy for selected solid tumors and mainly as a substitute for cisplatin in patients with chronic kidney disease. Although less frequent than cisplatin, carboplatin has been associated with nephrotoxicity expressed as tubular dysfunction and acute or chronic decline in the glomerular filtration rate (GFR). Carboplatin toxicity is highly dependent on GFR decline and systemic exposure. Thus, accurate dose adjustment based on GFR is the most important measure in the context of carboplatin nephrotoxicity.

## 1. Introduction

Carboplatin is a second-generation platinum-based chemotherapeutic agent developed to maintain the antitumor efficacy of cisplatin while reducing systemic toxicity, particularly nephrotoxicity [1]. It is widely used as a first-line therapy for selected solid tumors and as a substitute for cisplatin in patients deemed ineligible due to chronic kidney disease (CKD) and clinical frailty [2]. Despite carboplatin having been used for more than 35 years, several aspects of clinical manifestations, pathophysiology, and prophylaxis of carboplatin nephrotoxicity are not well established. Nephrotoxic events can be observed in up to 15% of patients treated with carboplatin, depending on the clinical context, chemotherapy regimen, and cumulative dose [3]. Hypomagnesemia is the most frequent renal adverse event, detected in up to 70% of patients [4]. In contrast, the reported incidence of acute kidney injury (AKI) directly attributable to carboplatin is variable. Clinical series and real-world studies have described AKI rates ranging from 2 to 7% [5,6,7], whereas some have cited estimates over 10% [3]. This variability likely reflects methodological differences, inconsistent AKI definitions, and a lack of causal adjudication rather than true differences in nephrotoxicity. Although less frequent than hypomagnesemia, reductions in glomerular filtration rate (GFR) carry greater clinical consequences, as they directly influence carboplatin dosing, cumulative hematologic toxicity, and overall treatment feasibility [8].

Carboplatin toxicity is highly dependent on systemic exposure, which is quantified by the area under the concentration–time curve (AUC) [9]. Inaccurate estimation of GFR, particularly when using the Cockcroft–Gault (CG) equation, frequently leads to GFR overestimation and carboplatin overdosing [8,10]. In this setting, dosing errors predominantly manifest as hematologic and clinical adverse events, which are more likely to be observed in older or sarcopenic patients [8,10]. Thus, in the carboplatin–kidney interaction, accurate dose adjustment based on GFR is at least as important as the intrinsic nephrotoxicity of the drug. Moreover, new equations to estimate GFR and a better knowledge of body composition allow more accurate carboplatin dosing, which is the major strategy to prevent carboplatin nephrotoxicity. In this review, we examine the renal complications associated with carboplatin, with particular emphasis on dose-adjustment strategies based on GFR, encompassing patients with CKD and those receiving dialysis, as well as practical considerations for the prevention and management of these complications.

## 2. Pathophysiology of Carboplatin Nephrotoxicity

### 2.1. AKI and GFR Decline

**Figure 1:**
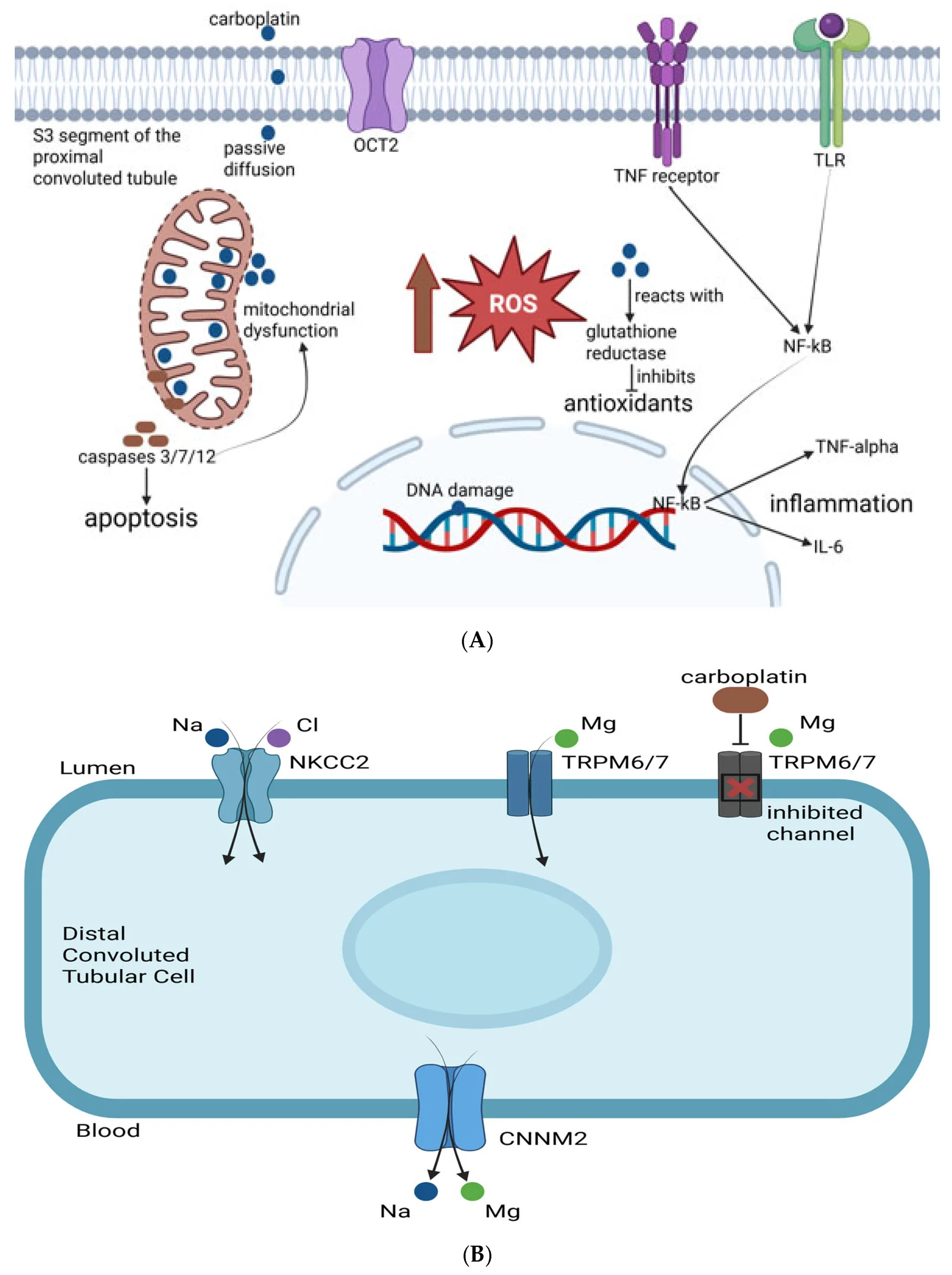
Potential pathophysiological mechanisms of carboplatin nephrotoxicity. (**A**) Tubular damage leading to AKI and persistent GFR decline. (**B**) Hypomagnesemia. Cl: chloride; CNNM2: cyclin M2 channel; IL-6: interleukin type 6; Mg: magnesium; Na: sodium; NF-κB: nuclear factor kappa B; NKCC2: Na-K-2Cl cotransporter; OCT2: organic cationic transporter type 2; ROS: reactive oxygen species; TNF-alpha: Tumor Necrosis Factor alpha; TLR: toll-like receptor; TRPM6/7: Transient Receptor Potential Melastatin types 6 and 7. Adapted from reference [20].

Carboplatin has lower nephrotoxicity than cisplatin. The absence of interaction between carboplatin and the human organic cationic transporter type 2 (OCT2) receptor reduces uptake of carboplatin into tubular cells and its consequent toxicity [11]. After entering the cell through passive diffusion, carboplatin has greater chemical stability and is therefore less reactive with DNA and has a weaker DNA-damaging effect than cisplatin [12]. Additionally, carboplatin does not bind avidly to plasma proteins, which augments urinary excretion and reduces its toxicity [13]. However, carboplatin can lead to AKI and chronic GFR decline at higher doses [14]. However, the molecular mechanisms responsible for carboplatin nephrotoxicity have not been described with the same consistency as those associated with cisplatin; a few potential pathways have been suggested in experimental studies, such as oxidative stress, mitochondrial dysfunction, and inflammation [15] (Figure 1A). Experimental studies have demonstrated that carboplatin suppresses the activity of antioxidant enzymes, such as renal tissue catalase, glutathione reductase, and heme oxygenase, leading to the liberation of free radicals and reactive oxygen species in the intratubular environment [15]. Carboplatin can also covalently form an adduct with glutathione by nucleophilic attack of its sulfhydryl group. This conjugate can be cleaved to a toxic intermediate, in addition to depleting glutathione per se and abrogating its antioxidant effect [16]. Additionally, the oxidative stress pathway activates caspases-3 and -7, which can lead to DNA damage, necrosis, and apoptosis of tubular cells, while caspase-12 activation can inhibit mitochondrial activity [17,18]. In addition to reactive oxygen species, reactive nitrogen species may also contribute to platinum-induced renal injury. Excessive activation of inducible nitric oxide synthase (iNOS) and subsequent nitric oxide dysregulation can promote nitrosative stress, mitochondrial dysfunction, and tubular epithelial injury, representing an additional mechanism involved in chemotherapy-associated nephrotoxicity [19].

There is increasing evidence supporting the role of inflammation in the pathophysiology of carboplatin nephrotoxicity. Boonmee et al. reported that carboplatin increases Transforming Growth Factor Beta 1 (TGF-β1) expression and primes macrophages for lipopolysaccharide (LPS), thereby enhancing production of proinflammatory cytokines such as Tumor Necrosis Factor alpha (TNF-alpha) and Interleukin (IL-6), which in turn activate Nuclear Factor kappa B (NF-κB) and the inflammatory cascade [21]. These pathophysiologic mechanisms can eventually translate into macroscopic tissue damage, with tubular necrosis and cortical atrophy demonstrated in histological investigations of the kidneys both in experimental models [22] and clinical reports [23].

In summary, although carboplatin and cisplatin share similar mechanisms of injury, the pathophysiology of carboplatin nephrotoxicity is less clearly understood and less frequently associated with loss of kidney function. A comparison of the main differences between carboplatin and cisplatin in terms of pharmacokinetic characteristics, clinical application, and nephrotoxic effects is depicted in Table A1.

### 2.2. Hypomagnesemia

The mechanism of carboplatin-induced hypomagnesemia remains unclear but is hypothesized to be similar to that of cisplatin [24]. Direct or indirect effects on the kidney and intestinal channel activity of Transient Receptor Potential Melastatin (TRPM) types 6/7 may partially explain this event. TRPM6/7 are channels highly permeable to magnesium (Mg) and calcium reabsorption in the intestine and distal convoluted tubule of the kidneys, playing an essential role in Mg homeostasis. Mutations, blockade, or inhibition of these channels by drugs can cause hypomagnesemia [25] (Figure 1B). Nevertheless, emerging data suggest that the correction of carboplatin-induced hypomagnesemia with Sodium-Glucose Linked Transporter type 2 (SGLT2) inhibitors occurs without interaction with TRPM6/7 [26].

## 3. Clinical Manifestations of Carboplatin Nephrotoxicity

Carboplatin nephrotoxicity is reported to be around 10–15% [3], and is more frequently manifested as hypomagnesemia and, to a lesser extent, kidney dysfunction (AKI, subacute kidney injury, and CKD) (Table A2). The main risks for these events are older age, dose per cycle (>800 mg/m^2^), cumulative dose (>2400 mg/m^2^), baseline GFR, and previous use of nephrotoxic drugs such as cisplatin [12,27].

### 3.1. AKI and GFR Decline

Early reports did not demonstrate a significant risk of AKI in carboplatin-based regimens, precluding mitigating strategies such as prophylactic hydration in this scenario [28]. More recently, AKI incidence rates up to 10% have been reported [28], probably influenced by closer vigilance.

Notably, carboplatin is the most frequently used platinum compound in patients with CKD, a factor that increases the risk of GFR decline. Although the risk of CKD progression is not clearly reported in most series, GFR decline of up to 20% compared to the baseline may occur in 8 to 22% of patients [29,30], but is not commonly associated with temporary or definitive treatment interruption.

AKI and persistent GFR decline during carboplatin therapy are frequently observed in the presence of concurrent kidney insults, such as hypovolemia, infection, and obstructive uropathy. In this scenario, nephrotoxicity directly attributable to carboplatin might be difficult to ascertain.

Recently, the draft of the KDIGO 2026 Clinical Practice Guideline for AKI [31] suggested the use of new biomarkers, besides creatinine, such as urinary NGAL, KIM1, and Tissue Inhibitor of Metalloproteinases type 2 [TIMP2] plus Insulin-like Growth Factor Binding Protein type 7 [IGFBP7] to diagnose, stratify, and assess the prognosis of AKI [32]. A few studies assessing adult patients with cancer exposed to platinum compounds demonstrated that these urinary NGAL [33], KIM-1 [34], and TIMP2/IGFBP7 [35] presented better sensitivity and were able to detect AKI earlier compared to serum creatinine. Although these studies assessed a small number of patients, these biomarkers could assist clinicians in tailoring protective hydration protocols or adjusting drug dosages to prevent kidney damage in patients treated with carboplatin.

### 3.2. Hypomagnesemia

Hypomagnesemia is the most frequent nephrotoxic effect in patients exposed to carboplatin, is usually mild, and rarely leads to delay or discontinuation of anticancer treatment. The main risk factors are high per-cycle dose (>282 mg/m^2^) and concomitant events such as vomiting or diarrhea, or exposure to medications such as proton-pump inhibitors, antibiotics, and other chemotherapeutic agents [4]. Although previous studies have reported an incidence of 10 to 15%, more recent series have reported higher frequencies of carboplatin-associated hypomagnesemia [4,36], likely due to increased vigilance. Liu et al. retrospectively evaluated 229 patients with advanced ovarian cancer treated with carboplatin (AUC = 5–6) and had a median of 14 (Interquartile Range [IQR]: 11–18) serum Mg measurements over the course of six (IQR: 5–8) cycles of therapy. The median frequency of hypomagnesemia (Mg < 1.8 mg/dL) was four (IQR: 1–7) occurrences; 84% (*n* = 192) of patients had at least one event, of which 97% were grade I (Common Toxicity Criteria [CTC]-based) (Mg > 1.2 mg/dL). Only one patient had a grade III event (Mg < 0.9 mg/dL) [36].

Gaughran et al. reported that at least one episode of hypomagnesemia was detected in 72% (*n* = 104) of patients from a cohort of 144 women with gynecological tumors receiving carboplatin (AUC = 5–6). Grade I and III/IV CTC-based events were observed in 70 and 11% of patients, respectively; 44% (*n =* 46) of them required intravenous or oral Mg replacement. The average duration of hypomagnesemia was 30 weeks, and 14% of events were not resolved at the end of follow-up, suggesting these patients are at risk of long-term renal toxicity [4].

These reports have brought attention to the risk of prolonged or permanent carboplatin-related hypomagnesemia. For this reason, monitoring of serum Mg levels should be incorporated in the routine practice of patients receiving carboplatin.

## 4. Carboplatin Dosing

Carboplatin clearance is linearly correlated with GFR and, when reduced, leads to decreased urinary elimination, prolonged half-life, increased systemic exposure, and a higher AUC. Consequently, reduced GFR is the main determinant of a higher risk of systemic toxicity, particularly thrombocytopenia [9]. Thus, accurately predicting carboplatin clearance is crucial for prescribing the correct dose of carboplatin and preventing the risk of both systemic and kidney-related toxicity.

### 4.1. The Calvert Equation

A landmark step in the carboplatin prescription journey occurred in 1989 when Calvert et al. published a formula to determine carboplatin dosing by assessing 31 patients with cancer who received 40 courses of carboplatin therapy [9]. Each patient’s GFR was measured by determining the plasma clearance of 51-chromium ethylenediaminetetraacetate (^51^Cr-EDTA). The total body clearance of carboplatin was equal to the GFR (mL/min) (renal clearance) plus 25 mL/min (non-renal clearance). The formula obtained was:

dose (mg) = target AUC ([mg/mL] × min) × (GFR + 25) (mL/min).

Due to limited access to mGFR in several countries, particularly in the United States, it has been substituted over the years for GFR estimating equations (eGFR) based on the serum level of creatinine (Cr) (eGFRCr), more frequently the CG equation, which estimates the Cr clearance [37]. This often leads to an overestimation of the carboplatin dose, and other equations have been assessed to overcome this issue, such as the MDRD and CKD-EPI equations, with diverse and discordant results over the years [38,39,40,41]. Most of these studies lacked mGFR as a reference when comparing the accuracy of different equations, and different assays were used to measure Cr (e.g., Jaffe vs. enzymatic, standardized vs. non-standardized), precluding the validation of GFR equations for carboplatin dosing for nearly 30 years after Calvert’s publication [42].

Notably, although the Calvert formula had been largely incorporated into clinical practice, the model can lead to large discrepancies between target and measured AUC, particularly in the case of high target AUCs [40]. This led to the development of alternative models, mathematically more robust, such as the creatinine-based Chatelut equation [41] and the creatinine and cystatin C-based Thomas equations [42], although these models were not incorporated into clinical practice worldwide.

### 4.2. Carboplatin Dosing Using Cr-Based GFR Estimating Equations

It has been unequivocally demonstrated that the CG equation is associated with larger errors in carboplatin dose than other eGFRCr equations [43,44]. A landmark study published in 2017 by Janowitz et al., including 2471 patients with solid tumors in England who were scheduled to receive carboplatin, assessed the percentage difference in carboplatin dose based on the Calvert equation using several eGFRCr equations compared to the dose calculated incorporating mGFR [44]. The CG equation had the highest error in the carboplatin dose, greater than 20% in more than 25% of patients. In contrast, only 19% and 14% of patients had errors greater than 20% when using CKD-EPICr and the new model (Janowitz or CamGFR equation), respectively. As limitations, this was a single-center study restricted to white patients, and the serum Cr assay was not standardized.

### 4.3. Carboplatin Dosing Using Cr and Cystatin C GFR Estimating Equations

A reference group in France collected pharmacokinetic data to demonstrate the impact of different GFR estimating equations based on Cr and cystatin C (Cys) on carboplatin dose. White-Koning et al. compared the predicted versus measured carboplatin clearance based on the drug’s serum level at multiple time points in 491 patients with cancer [42]. The Calvert equation based on CKD-EPICr-Cys was the best equation to predict carboplatin clearance, with the least bias, the lowest mean absolute percentage error (MPAE) (14.0 [95% CI: 12.9–15.2]%), and the lowest P20 (percentage of patients with MPAE over 20%) (23 [95% CI: 19–26]%) when compared to all other models used to predict GFR based on Cr alone: CKD-EPICr (28 [95% CI: 24–32]%), CG (34 [95% CI: 30–38]%), and MDRD (34 [95% CI: 30–38]%) formulas, incorporated into the Calvert equation. MPAE and P20 of CKD-EPICr-Cys were homogeneous in subgroups of age, sex, and body mass index (BMI). Although the study did not assess the impact on clinical outcomes and lacked mGFR, the authors demonstrated the benefit of incorporating Cys to estimate GFR in the Calvert equation using carboplatin clearance, a close proxy of mGFR.

Recently, Lees et al. performed a cross-sectional study including 1837 determinations of mGFR using single-point plasma clearance of iohexol within two years after cancer diagnosis in adult patients [45]. Concurrent measurements (same day) of Cr and Cys were collected within 30 days of mGFR and determined using standardized assays at the Karolinska University Hospital in Sweden. eGFRCr, eGFRCys, and eGFRCr-Cys based on the CKD-EPI and EKFC equations were calculated. The mean mGFR was 75 ± 30 mL/min, and the median recommended dose of carboplatin for a target AUC of 5 ranged from 320 mg (mGFR 30–44 mL/min) to 750 mg (mGFR 120 mL/min). Using eGFRCr would recommend an “overdose” of carboplatin in approximately 10–20% of individuals, with similar findings across sex, BMI, and metastatic disease subgroups. This was 3–4 times less common when using eGFRCr-Cys. Based on eGFRCys, an “underdose” of carboplatin was recommended in approximately 10–20% of individuals, with similar findings across subgroups, and was also 3–4 times less common when using eGFRCr-Cys. Although the study did not assess the impact of dose error on clinical outcomes, this is the most robust data demonstrating that incorporating Cys into GFR estimation reduces the chances of errors in carboplatin dosing.

### 4.4. Impact of Errors on Carboplatin Dosing on Clinical Outcomes

We are not aware of any studies demonstrating that improved accuracy of chemotherapy drug dosing, using an equation that better estimates mGFR, has directly led to an improvement in clinical outcomes in cancer populations, considering regimens based on carboplatin or other drugs. Emerging and promising data have been recently published. Lyu et al. retrospectively evaluated a cohort of 604 adult patients with non-small cell lung cancer who received cisplatin or carboplatin with an evaluation for sarcopenia, defined based on computed tomography scans using sex-specific cutoffs for skeletal muscle index [10]. Data on platinum-related adverse events (AEs), defined according to the CTC criteria, chemotherapy discontinuation, and mortality within 90 days of chemotherapy initiation were collected. In the subgroup of 565 patients receiving carboplatin (Calvert-calculated dose), those whose CG-based GFR dictated a carboplatin dose ≥25 mg higher than CKD-EPICr-based GFR were at significantly higher risk of chemotherapy discontinuation (hazard ratio [HR], 1.59; 95% CI, 1.06–2.38) and grade ≥2 AEs: anemia (hemoglobin < 10.0 g/dL) (HR: 1.59; 95% CI, 1.06–2.38); thrombocytopenia (platelet count < 75 × 10^9^/L) (HR: 2.15; 95% CI, 1.20–3.83) and Cr increase (1.5-fold increase in Cr from baseline) (HR: 1.82; 95% CI, 1.04–3.17).

Additionally, Khor et al. prospectively evaluated a group of 292 adult patients with solid tumors receiving carboplatin doses based on the Calvert equation at Massachusetts General Hospital [46]. Patients receiving “excess dose” (carboplatin dose based on CG equation >15% higher than the dose predicted by the CKD-EPICr-Cys) presented higher risk of grade 3 anemia (HR: 3.05; 95% CI, 1.35–6.91), thrombocytopenia (HR: 3,46; 95% CI, 1.01–11.8), AKI (HR: 6.50; 95% CI, 1.06–39.8), and mortality (HR: 3.16; 95% CI, 1.03–9.65) within 90 days of treatment initiation.

Although these studies lack mGFR, they suggest a detrimental impact of errors in carboplatin dosing on clinical outcomes, confirming the limitations of the CG equation compared to newly validated models and the clinical benefit of incorporating Cys in this scenario.

### 4.5. Impact of Body Composition on Carboplatin Dosing

Sarcopenic obesity is increasingly observed in patients with cancer [10]. Recently, body composition parameters have been shown to significantly impact GFR estimation [47].

In the study by Lyu et al., sarcopenia was detected in 167 (28%) patients. Patients with sarcopenia and BMI ≥ 25 kg/m^2^ (*n* = 67) presented an increased risk of grade ≥2 anemia (HR: 1.64; 95% CI, 1.17–2.29; *p* = 0.004), thrombocytopenia (HR: 2.25; 95% CI, 1.16–4.36; *p* = 0.016), and increased Cr (HR: 2.72; 95% CI, 1.45–5.13; *p* = 0.002) [10]. These data suggest an association between sarcopenic overweight/obesity and chemotherapy toxicity, which may be driven by GFR overestimation in this population. This supports the recommendations of current guidelines to prioritize the utilization of mGFR for carboplatin dose [48,49], particularly on the extremes of weight or suspected changes in body composition, as well as the incorporation of Cys in validated equations when mGFR is not available.

## 5. Dose Adjustment of Carboplatin in Chronic Kidney Disease

Recommendations for carboplatin prescribing in patients with CKD are summarized in Table A2 and outlined below, integrating the practical challenges of accurately determining the GFR input for the Calvert equation with dose considerations across levels of kidney function, according to the International Consensus Guideline for Anticancer Drug Dosing in Kidney Dysfunction (ADDIKD) [49]. Based on these guidelines, we adopted the following recommendations: (1) carboplatin dosing should be calculated using the Calvert formula rather than fixed percentage dose reductions across CKD categories [49]; (2) empiric reduction in the target AUC based solely on CKD stage is discouraged, as this may compromise antitumor efficacy without reliably reducing toxicity; (3) directly mGFR (reported in mL/min and not indexed to body surface area [BSA]) is the preferred GFR input for the Calvert formula, particularly in curative settings or when estimated GFR may be unreliable, including patients with extremes of body composition, sarcopenia, amputations, paraplegia, eGFR > 125 mL/min/1.73 m^2^, or eGFR ≤ 45 mL/min/1.73 m^2^ [49]; (4) recalculation of carboplatin dose at each cycle is generally unnecessary unless GFR changes by ≥20% or there is a significant alteration in clinical status; (5) dose adjustment according to GFR thresholds [49]. Additionally, we recommend: (6) BSA-adjusted eGFRCr-Cys models (mL/min) derived from validated equations when direct measurement is not feasible; (7) avoid CG Cr clearance because it is associated with larger errors and a higher frequency of adverse events [46]; (8) avoid race-based adjustments in GFR estimation in accordance with contemporary recommendations [45,50,51,52,53].

### Carboplatin Use in ESKD Patients

Carboplatin has a low molecular weight (~371 Da) and a relatively small volume of distribution, approximating the extracellular fluid compartment, which facilitates extracorporeal removal early after administration [54]. However, it undergoes time-dependent and largely irreversible protein binding, so only the early unbound fraction is meaningfully dialyzable [54]. Consequently, the effect of dialysis on carboplatin clearance is highly dependent on timing [55]. These considerations are particularly relevant in combination regimens, where concomitant agents may differ substantially in dialyzability, limiting the ability to optimize dialysis timing for carboplatin alone. The Calvert formula has been adapted for dialysis patients by assuming a GFR of zero [56]. This approach is appropriate when hemodialysis is performed within 12–24 h after infusion, while a meaningful unbound fraction remains dialyzable. Beyond this window, dialysis has limited impact on carboplatin clearance and may result in substantial systemic overexposure [56,57]. Pharmacokinetic modeling demonstrates that delayed hemodialysis may markedly increase systemic exposure, with significant AUC elevation in extreme scenarios despite dose adjustment [58]. Accordingly, expert recommendations support administering carboplatin on a non-dialysis day and scheduling hemodialysis 12–24 h later to enable partial extracorporeal clearance and better exposure control [55,56]. Data on carboplatin use in peritoneal dialysis are limited; available evidence suggests low dialytic clearance, supporting dosing as for GFR < 10 mL/min with close toxicity monitoring [59], as described in Table A2.

## 6. Management and Prevention of Adverse Events During Carboplatin Therapy

**Figure 2:**
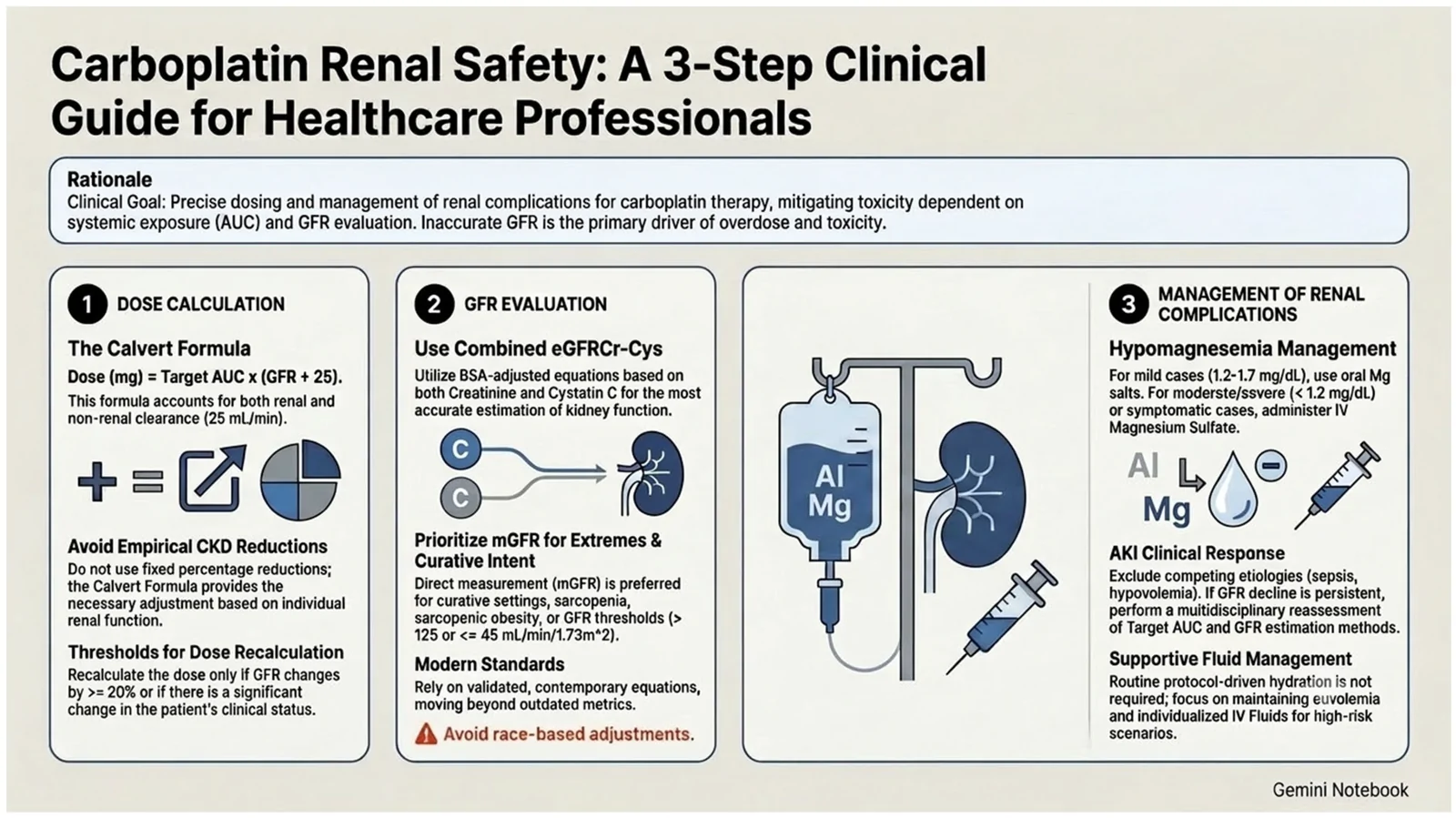
Schematic figure of carboplatin dosing, GFR evaluation, and management of renal complications. AKI: acute kidney injury; AUC: area under the curve; BSA: body surface area; CKD: chronic kidney disease; GFR: glomerular filtration rate; eGFRCr-Cys: Estimated Glomerular Filtration Rate based on the serum level of creatinine and cystatin C; mGFR: measured glomerular filtration rate; IV: intravenous; Mg: magnesium.

Overall, the prevention and management of adverse kidney events during carboplatin therapy rely less on prophylactic kidney strategies and more on accurate pharmacokinetic-guided dosing, appropriate monitoring, and coordinated treatment planning, particularly in patients with CKD and those undergoing dialysis (Table A3, Figure 2). Beyond pharmacokinetic optimization and supportive measures, future nephroprotective strategies may involve mechanism-driven modulation of inflammatory pathways. Given the complex role of inflammatory networks in tissue injury, targeted regulation of specific signaling pathways rather than nonspecific immunosuppression may represent a promising approach for reducing chemotherapy-associated renal damage [60].

### 6.1. Hypomagnesemia

Hypomagnesemia is the most frequent renal adverse event associated with carboplatin, and it is usually mild and cumulative. Therefore, periodic monitoring of serum Mg levels is recommended [4]. For asymptomatic or mild cases (serum Mg ≈ 1.2–1.7 mg/dL), oral Mg salts (e.g., Mg oxide) are frequently used due to convenience, although their absorption may be limited and gastrointestinal side effects (e.g., diarrhea) may occur. In moderate to severe hypomagnesemia (serum Mg < 1.2 mg/dL) or in symptomatic patients, intravenous Mg sulfate is the mainstay of treatment, with replenishment repeated as needed based on serial measurements to maintain serum Mg within an acceptable range [61]. In the absence of high-quality randomized evidence, routine prophylactic supplementation cannot be recommended for all carboplatin-treated patients.

Emerging experimental data [62] and early clinical reports suggest that SGLT2 inhibitors may attenuate platinum-associated renal Mg wasting [26,63]. A recent case described the resolution of acute carboplatin-induced hypomagnesemia with dapagliflozin, enabling the continuation of chemotherapy without intravenous supplementation [26]. A small case series reported improvement of refractory hypomagnesemia—including platinum-associated cases—in patients without diabetes treated with SGLT2 inhibitors [63]. However, the available evidence is limited to case reports and small series, with no prospective data on carboplatin-treated patients. Although promising, these findings are preliminary and do not support their routine use in clinical practice. In selected refractory cases, off-label therapy may be considered with careful monitoring for volume depletion and euglycemic ketoacidosis [64,65,66].

Finally, given the relatively low incidence of severe complications associated with hypomagnesemia, interruption of oncological therapy is generally unnecessary. In most cases, Mg replacement can be initiated while maintaining cancer treatment.

### 6.2. Acute Kidney Injury (AKI) and GFR Decline

While robust evidence supports hydration protocols and Mg supplementation for cisplatin [67], comparable data are lacking for carboplatin, and routine protocol-driven hydration or Mg supplementation cannot be recommended. Maintenance of euvolemia should be individualized, with selective use of intravenous fluids in higher-risk scenarios such as older age, lower baseline GFR, higher cumulative and per-cycle dose, and previous use of nephrotoxic drugs like cisplatin. AKI during carboplatin therapy should prompt an evaluation for competing etiologies before causal attribution, except in high-risk scenarios described above. Mild or transient increases in Cr do not mandate immediate discontinuation of carboplatin and should be managed by correcting reversible factors. Persistent or progressive GFR decline warrants reassessment of carboplatin dose, target AUC, and accurate measurement of GFR, particularly in older or sarcopenic patients. Consensus approaches, such as ADDIKD, recommend reassessing dosing and renal function when there is a clinically meaningful change in kidney function (e.g., ≥20% change in eGFR) [49]. In the setting of AKI during carboplatin therapy, decisions regarding treatment interruption should not rely solely on the degree of renal dysfunction. The overall oncological context must also be considered, including treatment intent (curative vs. palliative), availability of alternative therapeutic options, and the patient’s clinical response to therapy. In curative settings, greater efforts to preserve treatment intensity may be justified, whereas in palliative contexts, the risk-benefit considerations may differ. Accordingly, management decisions should be individualized and ideally made through shared decision-making involving the patient, oncologists, and nephrologists.

## 7. Conclusions

Carboplatin remains a cornerstone therapeutic agent widely used in the management of solid tumors, particularly for patients who are precluded from cisplatin therapy due to pre-existing nephrotoxicity or clinical frailty. While carboplatin exhibits a superior renal safety profile compared to cisplatin, kidney-related adverse events remain a distinct clinical concern that requires rigorous oversight. Future advancements in mitigating carboplatin-induced nephrotoxicity depend on a more robust elucidation of its underlying pathophysiology, alongside a precise characterization of the incidence of AKI and GFR decline in adult populations. Furthermore, optimizing patient outcomes will necessitate the development of targeted prophylactic strategies for high-risk cohorts.

## Data Availability

No new data were created or analyzed in this study. Data sharing is not applicable to this article.

## Abbreviations

The following abbreviations are used in this manuscript:

ADDIKD	Anticancer Drug Dosing in Kidney Dysfunction
AE	Adverse Event
AIN	Acute Interstitial Nephritis
AKI	Acute Kidney Injury
AUC	Area Under Concentration–time Curve
BMI	Body Mass Index
BSA	Body Surface Area
CG	Cockcroft–Gault equation
CKD	Chronic Kidney Disease
CKD-EPI	Chronic Kidney Disease Epidemiology Collaboration equation
CKD-EPICr	Equation based on creatinine
CKD-EPICys	Equation based on cystatin C
CKD-EPICr-Cys	Equation based on creatinine and cystatin C
CNS	Central Nervous System
Cr	Creatinine
^51^Cr-EDTA	^51^Chromium ethylenediaminetetraacetate
CT	Computed Tomography
CTC	Common Toxicity Criteria
Cys	Cystatin C
Da	Daltons
ECOG	Eastern Cooperative Oncology Group performance score
EKFC	European Kidney Function Consortium equation
ESKD	End Stage Kidney Disease
GFR	Glomerular Filtration Rate
eGFR	Estimated Glomerular Filtration Rate
eGFRCr	Estimated Glomerular Filtration Rate by creatinine
eGFRCys	Estimated Glomerular Filtration Rate by cystatin C
eGFRCr-Cys	Estimated Glomerular Filtration Rate by creatinine and cystatin C
mGFR	Measured Glomerular Filtration Rate
HR	Hazard Ratio
IGFBP7	Insulin-like Growth Factor Binding Protein type 7
IQR	Interquartile Range
IL	Interleukin
IV	Intravenous
KDIGO	Kidney Disease Improving Global Outcomes
KIM-1	Kidney Injury Molecule type 1
LPS	Lipopolysaccharide
MDRD	Modification of Diet in Renal Disease equation
Mg	Magnesium
MPAE	Mean Absolute Percentage Error
NGAL	Neutrophil Gelatinase-Associated Lipocalin
NF-κB	Nuclear Factor Kappa B
OCT2	Organ Cationic Transporter type 2
P20	Percentage over 20%
1-P30	Percentage of Accuracy within 30%
SGLT2	Sodium-Glucose Linked Transporter type 2
TIMP2	Tissue Inhibitor of Metalloproteinases type 2
Tc-DTPA	Tecnecium Diethylenetriaminepentaacetic Acid
TGF-β	Transforming Growth Factor Beta
TMA	Thrombotic Microangiopathy
TNF-alpha	Tumor Necrosis Factor alpha
TRPM6/7	Transient Receptor Potential Melastatin types 6 and 7

## Appendix A

**Table A1:** Major differences between cisplatin and carboplatin.

Characteristic [References]	Cisplatin	Carboplatin
Pharmacokinetics and Pharmacodinamics [68]	Interaction with OCT2 High adduction to DNA Half-life 13 min 25% of dose eliminated in 24 h, 90% renal, tubular secretion	No interaction with OCT2 100-fold lower adductivity to DNA Half-life 22 min 90% of dose eliminated in 24 h as unchanged drug, 100% renal, no tubular secretion
Pathophysiology of nephrotoxicity [69]	DNA damage Oxidative stress Mitochondrial damage Cell-cycle dysregulation Inflammation	Oxidative stress Mitochondrial damage Inflammation
Clinical Activity [70]	Standard for testicular, germ cell, bladder, head and neck tumors	Similar to cisplatin in non-small cell lung, gynecological (mainly ovarian) tumors
Adverse Events [68]	Higher nephrotoxicity Higher ototoxicity Higher emesis	Higher myelosuppression
Clinical Features of AKI [3,12,71]	Dose above 100 mg/m^2^ Prevalence up to 30% Typically 2 to 10 days after therapy, recovery after 2 to 4 weeks	Dose above 800 mg/m^2^ Prevalence up to 10% More delayed course, up to 64 days after therapy In association with other nephrotoxins or prior cisplatin exposure
Hypomagnesemia [67,72]	Prevalence 40 to 90% Half below 1.4 mg/dL Long-standing 10% symptomatic	Prevalence 10 to 15% 11% below 1.2 mg/dL Up to 30 weeks Rarely symptomatic
Other Clinical Features [73,74,75]	AIN Fanconi syndrome TMA	AIN (case reports) TMA (case reports)
Dose Adjustment [9,49,58]	GFR 45–60 mL/min/1.73 m^2^: decrease dose by 25% GFR < 45 mL/min/1.73 m^2^: discontinue drug	Calvert formula
Prophylaxis [67,76]	Hydration 2 L of saline Mg supplementation Mannitol not recommended	Routine saline hydration and Mg prophylactic supplementation not required

AIN: acute interstitial nephritis; AKI: acute kidney injury; GFR: glomerular filtration rate; Mg: magnesium; OCT2: organ cationic transporter type 2; TMA: thrombotic microangiopathy.

**Table A2:** Dose adjustment of carboplatin in CKD.

Dose Adjustment According to Kidney Function
Dose carboplatin using the Calvert formula; avoid empiric percentage dose reductions across CKD stages. Directly measured GFR (mL/min, not indexed to BSA) is preferred when: treatment intent is curative, extremes of body composition (sarcopenia, sarcopenic obesity, severe obesity), amputation or paraplegia, eGFR > 125 or ≤45 mL/min/1.73 m^2^. If estimating GFR: use BSA-adjusted eGFR (mL/min) derived from validated equations (e.g., CKD-EPI 2021, EKFC); use combined Cr-Cys equations whenever possible; avoid CG; avoid race-based adjustments. Dose adjustment according to GFR thresholds: ➢ GFR ≥ 60 mL/min/1.73 m^2^: target AUC using the Calvert formula. Capping GFR thresholds is not recommended, as it may reduce therapeutic efficacy without reducing toxicity; ➢ GFR 30–59 mL/min/1.73 m^2^: target AUC using the Calvert formula. Increased risk of hematologic toxicity warrants enhanced monitoring, particularly in frail or heavily pretreated patients; ➢ GFR 15–29 mL/min/1.73 m^2^: dose determination should preferably use mGFR. Given the higher risk of adverse events, multidisciplinary discussion is recommended; ➢ GFR < 15 mL/min/1.73 m^2^ (not receiving kidney replacement therapy): carboplatin dosing should be managed by a multidisciplinary team involving oncology/hematology, nephrology, and/or clinical pharmacology, as evidence to guide safe dosing in this population remains limited.
**Carboplatin in ESKD and Dialysis**
In hemodialysis patients, dose using the Calvert formula assuming GFR = 0 Administer carboplatin on a non-dialysis day and schedule hemodialysis 12–24 h after infusion to allow partial extracorporeal clearance of unbound drug Delayed dialysis (>24 h) may significantly increase systemic exposure In peritoneal dialysis, dialytic clearance is minimal; dose conservatively (similar to GFR < 10 mL/min) with close toxicity monitoring All dosing decisions in ESKD should involve multidisciplinary discussion

AUC: area under curve; BSA: body surface area; CG: Cockcroft–Gault equation; CKD: chronic kidney disease; CKD-EPI: Chronic Kidney Disease Epidemiology Collaboration equation; Cr: creatinine; Cys: cystatin C; EKFC: European Kidney Function Consortium equation; ESKD: end-stage kidney disease; GFR: glomerular filtration rate; eGFR: Estimated Glomerular Filtration Rate.

**Table A3:** Practical recommendations for prevention and management of AE during carboplatin therapy.

General Principles
Renal AE associated with carboplatin is uncommon and usually multifactorial. Prevention should prioritize accurate pharmacokinetic-guided dosing and monitoring rather than routine renal prophylaxis. Management decisions should integrate renal function changes with oncologic context (curative vs. palliative intent, treatment response, alternative options).
**Hypomagnesemia**
Monitor serum Mg periodically, particularly during prolonged therapy and in high-risk patients (baseline hypomagnesemia, gastrointestinal losses, ostomies, diuretics, calcineurin inhibitors, or previous platin exposure. Mild hypomagnesemia (≈1.2–1.7 mg/dL): oral Mg supplementation is appropriate, recognizing limited bioavailability and gastrointestinal intolerance. Moderate/severe hypomagnesemia (<1.2 mg/dL) or symptomatic cases: intravenous Mg sulfate with serial monitoring. Routine prophylactic Mg supplementation is not recommended in the absence of prior deficiency or high-risk features. Interruption of carboplatin is rarely necessary for hypomagnesemia. Replacement therapy can usually be administered while maintaining oncologic treatment.
**Supportive Fluid Management**
Routine protocol-driven hydration is not required for carboplatin (unlike cisplatin). Maintain euvolemia; avoid both hypovolemia and unnecessary fluid overload. Consider selective intravenous fluid administration in higher-risk scenarios: borderline GFR, significant gastrointestinal losses, elderly or frail patients, high-intensity or cumulative carboplatin regimens (e.g., >800 mg/m^2^).
**AKI**
Evaluate alternative causes of AKI (sepsis, hypovolemia, contrast exposure, nephrotoxic medications) before attributing causality to carboplatin. Mild or transient increases in serum creatinine do not mandate automatic discontinuation. Correct reversible factors (volume status, medications) and repeat kidney function testing. Persistent or progressive AKI (CTC grade ≥ 2) should prompt reassessment of: carboplatin dose and target AUC, method of GFR estimation, concomitant nephrotoxic exposures. Consider mGFR in patients with dynamic renal impairment or suspected GFR misclassification (e.g., sarcopenia). Monitor for infusion-related hypersensitivity reactions, which may signal immune-mediated injury such as AIN. Treatment interruption decisions should be individualized and ideally made jointly by oncology and nephrology teams.

AE: adverse event; AIN: acute interstitial nephritis; AKI: acute kidney injury; AUC: area under concentration–time curve; CTC: common toxicity criteria; GFR: glomerular filtration rate; mGFR: measured glomerular filtration rate; Mg: magnesium.
